# Risk of psychosis among migrants to the Netherlands by time since arrival

**DOI:** 10.1017/S0033291722001192

**Published:** 2023-07

**Authors:** Fabian Termorshuizen, Jean-Paul Selten

**Affiliations:** 1 Rivierduinen Institute for Mental Health Care, Sandifortdreef 19, 2333 ZZ Leiden, The Netherlands; 2 Department of Psychiatry and Neuropsychology, School for Mental Health and Neuroscience, Maastricht University Medical Centre, P.O. Box 616, 6200 MD Maastricht, The Netherlands

**Keywords:** Antipsychotics, epidemiology, healthy migrant effect, migration, psychotic disorder

## Abstract

**Background:**

The high risk of psychosis among migrants is often attributed to social stressors in the host country. We examined whether the relative risk of psychosis among migrants is low on arrival and increases thereafter.

**Methods:**

In this cohort study, first-generation immigrants to the Netherlands, aged 10 years and older (*N* = 1 281 678), were matched by birth year and sex to 2 542 313 native-born Dutch controls. The first occurrence of psychosis after arrival was established using data on dispensing of antipsychotic medication (APM) (during 2006–2017) and on insurance claims for treatment of psychosis (2011–2016). The Incidence Rate Ratios (IRRs) for migrants compared to controls were estimated by year since arrival.

**Results:**

The IRR of APM was 0.22 (95% CI 0.21–0.24) in the year of arrival (‘year 1’) and increased gradually to 1.39 (1.19–1.62) after 10 or more years. The IRR of an insurance claim increased from 0.57 (0.51–0.62) to 1.87 (1.38–2.55) in year 5. Among migrants from sub-Saharan Africa, the IRR of an insurance claim was already high in year 1 [2.46 (1.95–3.11)], especially when aged 10–20 years at arrival [6.09 (2.93–12.64)]. Among migrants from other non-Western countries, the IRR was already significantly increased in year 2 [1.28 (1.03–1.59)].

**Conclusions:**

The relative risk of psychosis among migrants was generally low at arrival and increased thereafter. The increased IRRs in the early years after arrival among those from non-Western countries indicate that for these groups certain risk factors are already relevant shortly after arrival.

## Introduction

There have been consistent reports of an increased incidence of affective and non-affective psychotic disorders among migrants to Western Europe, especially when they come from a developing country (Bourque, van der Ven, & Malla, 2011; Selten, van der Ven, & Termorshuizen, 2020). This despite the premise that migrants constitute a positive selection from the population in the country of origin, the so-called healthy migrant effect (HME) (Dhadda & Greene, 2018; Hamilton, 2015; Vang, Sigouin, Flenon, & Gagnon, 2017). Furthermore, selective migration of those at high risk for developing a psychotic disorder does not appear to explain the increased incidence among immigrants (Lundberg, Cantor-Graae, Kabakyenga, Rukundo, & Ostergren, 2004; Selten, Cantor-Graae, Slaets, & Kahn, 2002; van der Ven et al., 2015). As there is no evidence of an increased incidence of psychosis in several regions of origin (Bhugra et al., 1996; Selten et al., 2005), exposure to social stressors in the host country may explain at least a part of this phenomenon (Dykxhoorn & Kirkbride, 2019; Selten & Cantor-Graae, 2005). These stressors probably concern difficulties in obtaining access to mainstream society and are likely to become more intense when success fails to materialise (Selten & Cantor-Graae, 2005). If this is true, then the risk of psychosis among immigrants might be lower than that of the native-born population shortly after arrival, and the relative risk might increase with time thereafter. This was found for general mental health problems and depression (Rivera, Casal, & Currais, 2016; Wu & Schimmele, 2005). Very few studies have examined the time interval between arrival and the onset of psychosis. The study by Ødegaard in 1932 among Norwegian migrants to Minnesota, USA, reported an interval of at least 5 years and a median duration of 10 years until the onset of psychosis (Odegaard, 1932). A more recent study in Malmö (Sweden) found a mean duration between arrival and first contact for schizophrenia-like psychosis of 9 years (Zolkowska, Cantor-Graae, & McNeil, 2001). No studies have investigated the relative risk of psychosis among migrants compared to the native-born population by time since arrival. We analysed registered data on dispensed antipsychotic medication (APM) and insurance claims for psychiatric treatment for a psychotic disorder as proxies for psychosis for all migrants together, and then by region of birth and age at arrival. We hypothesised that the relative risk of psychosis among migrants compared to native-born persons is lower than 1.0 at the time of arrival and gradually increases with time thereafter.

## Methods

### Data sources

The population register managed by Statistics Netherlands (Centraal Bureau voor de Statistiek, CBS) records information on core demographic variables for all legal residents in the Netherlands. The data are derived from municipal registries, not from voluntary door-to-door surveys. Registration with municipal authorities is compulsory in the Netherlands and a prerequisite for essential documents (e.g. residence or work permit) and aid (e.g. income support).

The second database, run by the Health Care Institute Netherlands (Zorginstituut Nederland, ZiN), contains information on dispensed medication reimbursed by all health-insurance companies during the period 2006–2017. Citizens are obliged by law to have medical insurance. Asylum seekers receive a health insurance shortly after arrival. Since there is no distinction between public and private health-insurance companies, the results of our study are not influenced by the selection of people who can afford health insurance. This database records information on drugs dispensed to outpatients and to patients in nursing homes, but not on drugs dispensed during in-patient treatment. For a particular calendar year and a given individual, the first four positions of the ATC code are mentioned only once. Thus, it is possible to establish whether a person had medication dispensed in a certain calendar year and for which classes of drugs (e.g. A10A: insulins and analogs; N05A: APM).

The third database is the register of the so-called Diagnosis Treatment Combinations (DTCs) (Dutch: Diagnose Behandel Combinatie, DBC) of the Dutch Healthcare Authority (Nederlandse Zorgautoriteit, NZa). The NZa collects information from all health-insurance companies in the Netherlands. A DTC is an insurance claim, that has to be renewed each year, based on codes for diagnosis and treatment by a medical specialist, with accompanying starting- and end-dates. For the present study, DTC data from all Mental Health Care Institutes in the Netherlands were available for the calendar years 2011–2016.

Staff of Statistics Netherlands linked the information from the three databases, using the civil identification number, unique for each citizen.

### Ethical standards

Dutch privacy laws allow the use of personal (health care) data for medical-scientific research without informed consent, provided that the results of the analysis cannot be traced to an individual. Consequently, the postal code and the civil identification number were removed from the files used in this study.

### Study group

All first-generation migrants with a registered date of immigration in the period 2006–2017 and with an legal address of residence were selected from the population registry (*N* = 1 434 466). Then all native-born residents were selected to constitute the pool of control persons with the exclusion of second-generation migrants (i.e. born in the Netherlands to one or two foreign-born parents). They were matched on an individual level by sex and birth year in 5-year categories. For each migrant there were at least two controls. After checking for overlap in follow-up time (that is, the control person was alive and residing in the Netherlands at the date of immigration of the matched migrant), there was information on 21 043 migrants with one control and 1 412 719 migrants with at least two controls. Then migrants aged 10 years or older at the time of arrival were selected. This resulted in a study group of *N* = 1 281 678 migrants and *N* = 2 542 313 personally matched controls, the ‘reference population’ (*N* = 21 043 migrants with 1 control, *N* = 1 260 635 with 2 controls).

### Follow-up

As the data on medication were registered for each calendar year, this information was broken down accordingly. For both the migrant and the matched control(s), the follow-up started in the calendar year of arrival (‘year 1’). The maximum follow-up was 12 years (arrival in 2006 and alive and living in the Netherlands in 2017). For migrants in the calendar year of arrival, only the days after arrival were taken into account for the calculation of follow-up time in that year. For controls, when alive and living in the Netherlands at January 1st, all days of that calendar year were included in the calculation of follow-up time. Follow-up ended when the migrant or one of the matched controls died or emigrated. After the first dispensing of APM or the first diagnosis of psychosis from a DTC, the person was excluded from the analysis.

### Outcomes: antipsychotic medication and diagnosis treatment combinations

For each calendar year of follow-up, record linkage with the ZiN data yielded a variable that indicated the dispensing (yes/no) of an APM. The N05A codes N05AH04 (Quetiapine), -C01 (Periciazine), -D08 (Droperidol), -D05 (Pipamperone), -A02 (Levomepromazine), and -N (Lithium) were excluded because these drugs are often prescribed for other disorders. The first dispensing in or after the calendar year of arrival was considered the incident Dispensing of an APM (IDAP, hence in text: ‘Dispensing’), for both migrants and their matched controls. Information on drugs dispensed before this calendar year was not available for migrants and was not considered for controls in the primary analysis.

For migrants who arrived in the period 2011–2016 and their controls, record linkage with the DTC data on utilisation of health services was possible for a maximum of 6 follow-up years. For each calendar year of follow-up, a variable was added to the analysis file to indicate whether the subject had undergone psychiatric treatment for a broadly defined psychotic disorder. That is, all DSM-IV codes indicative of an affective or non-affective psychotic disorder, either as main or as second diagnosis, were selected (295.xx, 297.1/3, 298.8/9, 296.24/34/44/54/04/64). Similar to the definition of Dispensing, the first registration of a DTC in or after the calendar year of arrival was regarded as the Incident moment for a DTC for psychosis (IDTC, hence in text: ‘Treatment’). Data on DTCs before this calendar year were not available for migrants and were not considered for controls in the primary analysis.

### Statistical analysis

The dataset was aggregated by migrant/control status, age at arrival, year of follow-up, and country/region of birth. For each stratum, the numbers of Dispensings, Treatments, and days of follow-up were calculated. The numbers of Dispensings and Treatments, both divided by the number of days of follow-up, were used as the outcome in two separate Poisson regression analyses.

First, migrant status (all migrants *v.* their matched controls), number of years after arrival as categorised variable (1 up to 10 or more), and terms for interaction of (migrant status *v.* number of years) were included as independent variables. A significant interaction indicates that time since arrival affects the Incidence Rate Ratio (IRR) for migrants compared to controls. The term IRR does apply here, because the incidence figures are based on first cases within the time window of observation. Given the chronic nature of psychotic disorder it is likely that many Dispensings and Treatments did not occur for the first time in the life of controls in the year of arrival of their matched migrant. As for migrants, however, the situation may be different. Since psychosis renders the complex act of migration difficult, it is likely that only few migrants have a history of psychosis in their country of origin. Thus, in order to obtain estimates that may better approximate the life-time incidence of psychosis, we conducted a sensitivity analysis on Dispensings and Treatments, respectively, from which we excluded controls with a registered dispensing of APM or a DTC for psychosis in the calendar year prior to the year of arrival of their matched migrant. This was not possible for controls matched to migrants who arrived in 2006 (with reference to the analysis on Dispensing) and in 2011 (analysis on Treatment).

Statistics Netherlands distinguishes, somewhat arbitrarily, between Western countries (Europe, countries of the former Soviet Union with a predominantly Christian religion, the USA, Canada, Australia, New-Zealand), and non-Western countries (all other countries). Separate analyses were performed for Western and for non-Western migrants and for two migrant groups with a recent migration history: migrants from Eastern Europe (that is, formerly communist countries) and migrants from sub-Saharan Africa.

Secondly, in order to examine whether the time trends in the IRR differed by age at arrival, the models for Dispensings and Treatments were extended to include age at arrival (10–20, 20–40, 40–60, >60 years). To make meaningful analyses possible for small subgroups by age and region of origin, time since arrival was classified as 1, 2–3, 4–6, and >6 years.

APM is often prescribed off-label to people without a psychotic disorder (Carton et al., 2015). In order to assess the possible over- or under-estimation of the IRRs when Dispensing was used as proxy for psychosis, we estimated the proportion of persons with Treatments among those with Dispensings, and the proportion of persons with Dispensings among those without Treatments. This was done by year since arrival and for migrants and their controls separately. These proportions were used to re-calculate the crude incidence figures for Dispensings and associated IRRs (see online Supplementary results and Supplementary Tables S8 and S9*a*–*d*).

Data preparation, record linkage and estimation of crude rates were performed using SPSS version 25.0. The Poisson regression analysis was conducted using STATA version 16.0.

## Results

### Description

Most migrants (73.3%) were 20–40 years old at arrival (online Supplementary Table S1). Online Supplementary Table S2 shows the numbers of migrants and controls who arrived in the years 2011–2016, and, thus, for whom data on DTCs were available.

### Dispensings and treatments, all migrants

**Fig. 1.**
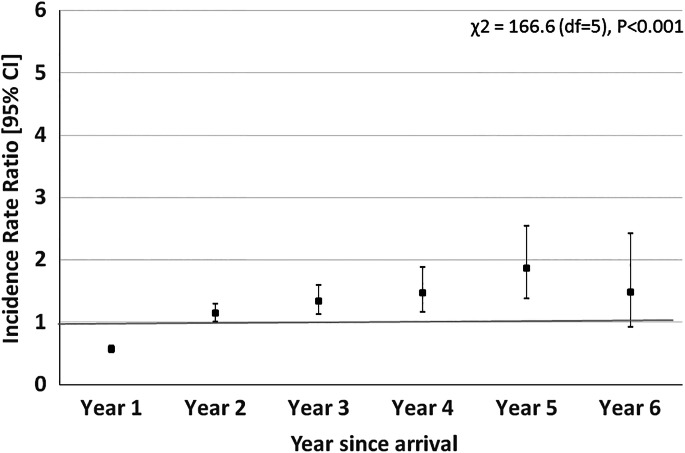
Migrants to the Netherlands (all).

Table 1 gives the numbers of persons, person-days of follow-up, Dispensings, and Treatments, and incidence rates for migrants and controls, by year since arrival. In the year of arrival (‘year 1’), the rate of Dispensings for migrants was significantly lower than that for controls [IRR = 0.22, (0.21–0.24)]. In the years thereafter, an increasing trend in the IRR was found to 1.39 [1.19–1.62] after 10 or more years. The differences in IRR between the years of follow-up [that is, the terms for interaction of (year × migrant)] were statistically significant (*p* < 0.001). For Treatment as outcome, a similar trend was found, with a low IRR of 0.57 [0.51–0.62] in the year of arrival and increasing IRRs to values above 1 in the years thereafter. However, the IRR was already significantly increased in ‘year 2’ [IRR = 1.14 (1.01–1.30)], suggesting that the IRR increases already in the early years after arrival (Fig. 1).

**Table 1.** Migrants to the Netherlands v. native-born controls (=Reference population): numbers of persons, numbers of Incident Dispensings of Antipsychotic Medication (IDAPs, 2006–2017) and of Incident Diagnosis Treatment Combinations for psychosis (IDTCs, 2011–2016), rates (number/10 000 person-years), and Incidence Rate Ratios (IRRs), by number of years since arrival

Years since arrival	Reference population			Rate (/10 000 person- years)	Migrants	*N* days	Number of IDAPs/IDCTs	Rate (/10 000 person- years)	IRR [95%–CI]
	*N* persons	*N* days	Number of IDAPs/IDCTs		*N* persons				
IDAP									
1	2 542 313	927 530 994	18 801	74.0	1 281 678	210 297 792	955	16.6	0.22 [0.21–0.24]
2	2 014 123	733 209 302	4407	21.9	1 021 490	329 452 318	1324	14.7	0.67 [0.63–0.71]
3	1 333 295	485 415 610	2590	19.5	676 118	225 269 708	972	15.7	0.81 [0.75–0.87]
4	939 673	342 154 001	1760	18.8	476 349	161 895 577	766	17.3	0.92 [0.85–1.00]
5	692 474	252 196 733	1250	18.1	350 643	121 030 296	568	17.1	0.95 [0.86–1.05]
6	525 618	191 458 099	962	18.3	265 881	92 807 740	504	19.8	1.08 [0.97–1.20]
7	404 172	147 267 141	727	18.0	204 138	71 829 248	377	19.2	1.06 [0.94–1.20]
8	299 811	109 218 912	507	16.9	151 176	53 434 885	297	20.3	1.20 [1.04–1.38]
9	215 010	78 345 432	375	17.5	108 209	38 508 994	219	20.8	1.19 [1.01–1.40]
≥10	257 845	93 992 963	389	15.1	129 423	46 324 431	266	21.0	1.39 [1.19–1.62]
							Year 1–10: χ^2^, df, *p* value		1537.7, 9, <0.001
IDTC									
1	1 410 003	514 559 564	3734	26.5	711 844	116 051 880	477	15.0	0.57 [0.51–0.62]
2	988 713	360 084 306	737	7.5	500 001	159 317 085	373	8.5	1.14 [1.01–1.30]
3	562 261	204 706 790	334	6.0	284 196	94 314 627	206	8.0	1.34 [1.13–1.59]
4	329 271	119 926 160	160	4.9	166 328	56 483 038	111	7.2	1.47 [1.16–1.88]
5	185 522	67 605 881	86	4.6	93 637	32 319 596	77	8.7	1.87 [1.38–2.55]
6	85 351	31 149 225	39	4.6	43 011	15 035 636	28	6.8	1.49 [0.92–2.42]
							Year 1-6: χ2, df, *p* value		166.6, 5, <0.001

After exclusion of controls with a dispensed APM in the calendar year prior to the year of arrival of their matched migrant, the IRR of Dispensing in year 1 became higher and the increasing trend less pronounced (online Supplementary Table S3). After exclusion of controls with a DTC for psychosis in this year, the IRR of Treatment was already significantly higher than 1.00 in the year of arrival [IRR = 1.41 (1.27–1.56)] and there was no longer a clear trend in the years thereafter (online Supplementary Fig. S1 and Supplementary Table S3).

### Treatments by region of origin

**Fig. 2.**
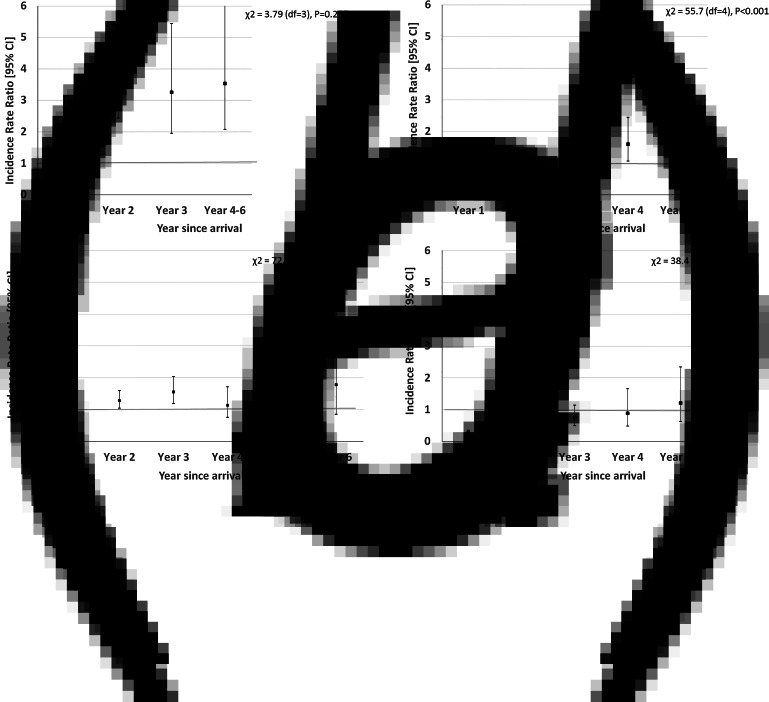
(*a*) Migrants from sub-Saharan Africa. (*b*) Migrants from other non-Western^(1)^ countries. (*c*) Migrants from Eastern Europe. (*d*) Migrants from other Western^(2)^ countries. Incidence Rate Ratio (IRR) of Incident Diagnosis Treatment Combinations (IDTCs) for psychosis among migrants to the Netherlands (2011–2016) compared to native-born controls (=Reference population), by year since arrival. (1) Non-Western: all countries not mentioned under (2). (2) Western: Europe, countries of the former Soviet Union with a predominantly Christian religion, the USA, Canada, Australia, New-Zealand.

Data were then analysed by region of origin (Fig. 2 and online Supplementary Table S4). Comparatively low rates of Treatments in the year of arrival and increasing trends in the IRR in the years thereafter were found for non-Western migrants from regions other than sub-Saharan Africa, for migrants from Eastern-Europe and for migrants from other Western countries (Fig. 2*b*–*d*). For migrants from other non-Western countries, the IRR was already significantly increased in ‘year 2’ [IRR = 1.28 (1.03–1.59)] (Fig. 2*b*). For migrants from sub-Sahara Africa, the IRR of Treatment was already significantly increased in the year of arrival [2.46 (1.95–3.11)] without a trend thereafter (Fig. 2*a*).

After exclusion of prevalent cases among controls, the IRRs appeared to be already higher than one in the year of arrival and without evidence of a time trend thereafter for those from other non-Western countries and Eastern-Europe (online Supplementary Table S5). For Western migrants from other countries than Eastern Europe, low values for the IRR were found in the years after arrival (year 1–3) and no IRR significantly higher than 1.00 in the years thereafter (year 5–6) could be established. For migrants from sub-Saharan Africa, the IRR was significantly higher than 1.00 in all years of follow-up, irrespective of in- or exclusion of prevalent cases among controls.

### Dispensings and treatments by age, all migrants

The IRRs of Dispensing among subjects in the age groups younger than 60 years were significantly lower than 1.00 in the year of arrival and increased to values significantly higher than 1.00 after 6 years (Table 2). After exclusion of prevalent cases among controls, this trend became less pronounced, but the differences between the years were still statistically significant (online Supplementary Table S6). Owing to small numbers, the age group 60 years and older was excluded.

**Table 2.** Migrants to the Netherlands v. native-born controls (=Reference population): see Table 1, by number of years since arrival and age

Age at arrival*	Reference population		Rate (/10 000 person-years)	Migrants *N* persons	Number of IDAPs/IDTCs	Rate (/10 000 person-years)	IRR [95%–CI]
Years since arrival	*N* persons	Number of IDAPs/IDTCs					
IDAP 10–20 years 1	339 097	2322	68.5	169 811	106	14.7	0.21 [0.18–0.26]
2–3	464 468	940	20.3	234 126	278	12.8	0.63 [0.55–0.72]
4–6	314 426	602	19.2	158 718	225	15.0	0.78 [0.67–0.91]
>6	165 696	322	19.5	83 550	189	23.4	1.20 [1.00–1.44]
					Year 1–10: χ^2^, *df*, *p* value		174.7, 3, <0.001
20–40 years 1	1 858 177	12 922	69.6	939 307	549	13.0	0.19 [0.17–0.20]
2–3	2 420 636	4951	20.5	1 230 485	1483	13.6	0.66 [0.63–0.70]
4–6	1 530 352	2653	17.4	776 846	1206	16.5	0.95 [0.89–1.02]
>6	839 798	1291	15.4	423 700	744	18.1	1.17 [1.07–1.29]
					Year 1–10: χ^2^, *df*, *p* value		1103.8, 3, <0.001
40–60 years 1	314 237	3169	100.9	157 124	188	25.3	0.25 [0.22–0.29]
2–3	421 775	868	20.6	212 614	400	20.6	1.00 [0.89–1.13]
4–6	285 586	532	18.7	143 615	327	24.0	1.29 [1.12–1.48]
>6	157 942	270	17.1	78 982	181	23.5	1.37 [1.14–1.66]
		,			Year 1–10: χ^2^, *df*, *p* value		330.8, 3, <0.001
IDTC 10–20 years 1	195 271	125	6.4	97 677	43	10.6	1.65 [1.17–2.34]
2–3	222 393	98	4.4	111 240	68	6.6	1.50 [1.10–2.04]
4–6	90 540	44	4.9	45 258	35	8.2	1.69 [1.09–2.64]
					Year 1–6: χ^2^, *df*, *p* value		0.26, 2, 0.876
20–40 years 1	1 026 310	2876	28.0	519 935	342	14.7	0.52 [0.47–0.59]
2–3	1 116 691	836	7.5	566 750	412	8.3	1.11 [0.98–1.25]
4–6	421 861	212	5.0	213 818	143	7.1	1.42 [1.15–1.75]
					Year 1–6: χ^2^, df, *p* value		111.6, 2, <0.001
40–60 years 1	171 571	686	40.0	85 788	83	20.4	0.51 [0.41–0.64]
2–3	193 238	129	6.7	96 854	91	10.3	1.55 [1.18–2.02]
4–6	79 926	25	3.1	39 991	33	8.7	2.78 [1.65–4.67]
					Year 1–6: χ^2^, *df*, *p* value		57.6, 2, <0.001

The IRRs of Treatment showed a similar trend among those aged 20–40 and 40–60 years, but with estimates (borderline) significantly higher than 1.00 already 2–3 years after arrival (Table 2). The use of Treatments as outcome resulted in higher estimates and higher estimates at an earlier stage after arrival than the use of Dispensings. Among those aged 10–20 years, a high IRR of Treatment was already found in the first year [1.65 (1.17–2.34)], and without a time trend therafter. After exclusion of prevalent cases among controls, the IRRs were already significantly higher than 1.00 in all age groups in the year of arrival and without significant differences between the years thereafter indicative of an increasing trend (online Supplementary Table S6).

### Treatments by age and region of origin

Extremely high IRRs of Treatment were found among migrants from sub-Saharan Africa aged 10–20 years in all years following arrival (Table 3 and online Supplementary Table S7).

**Table 3.** Migrants to the Netherlands v. native-born controls (=Reference population): Rates for migrants (number/10 000 person-years) and Incidence Rate Ratios (IRRs) of Incident Diagnosis Treatment Combinations (IDTCs, 2011–2016), by number of years since arrival, age, and region of origin

Age at arrival*	10–20 year	IRR [95%–CI]	20–40 year	IRR [95%–CI]	40–60 year	IRR [95%–CI]
Region of origin	Rate (/10 000 person-years)		Rate (/10 000 person-years)		Rate (/10 000 person-years)	
Years since arrival						
Non-Western migrants: other 1	9.9	1.63 [0.94–2.85]	13.7	0.50 [0.41–0.61]	25.2	0.64 [0.45–0.90]
2–3	7.4	2.18 [1.30–3.66]	8.4	1.13 [0.93–1.38]	14.5	2.84 [1.81–4.46]
4–6	8.0	1.45 [0.73–2.87]	6.9	1.35 [0.95–1.91]	10.7	2.91 [1.29–6.56]
Year 1–6: χ^2^, *df*, *p* value		1.02, 2, 0.601		42.2, 2, <0.001		31.4, 2, 0.001
Non-Western migrants: Sub-Saharan Africa 1	32.9	6.09 [2.93–12.64]	62.4	2.32 [1.78–3.04]	48.9	2.03 [0.99–4.15]
2–3	17.8	8.27 [3.38–20.22]	23.4	3.03 [2.12–4.34]	13.0	2.50 [0.84–7.45]
4–6	16.8	10.27 [2.25–46.86]	19.0	2.92 [1.57–5.43]	7.4	2.08 [0.29–14.75]
Year 1–6: χ^2^, *df*, *p* value		0.50, 2, 0.778		1.52, 2, 0.468		0.10, 2, 0.950
Western migrants: Eastern Europe 1	8.7	1.41 [0.61–3.26]	14.3	0.48 [0.39–0.59]	16.9	0.39 [0.25–0.61]
2–3	3.2	0.62 [0.27–1.44]	8.3	1.11 [0.90–1.38]	8.7	1.08 [0.67–1.76]
4–6	6.3	1.16 [0.43–3.14]	7.5	1.42 [0.99–2.04]	10.5	3.88 [1.55–9.73]
Year 1–6: χ^2^, *df*, *p* value		1.98, 2, 0.371		41.9, 2, <0.001		23.0, 2, <0.001
Western migrants: other 1	1.0	0.13 [0.02–0.97]	7.1	0.26 [0.19–0.35]	12.1	0.30 [0.17–0.53]
2–3	2.8	0.46 [0.21–0.98]	4.9	0.64 [0.49–0.84]	6.5	0.89 [0.50–1.58]
4–6	5.3	1.03 [0.36–2.98]	3.8	0.92 [0.53–1.59]	4.1	1.45 [0.41–5.13]
Year 1–6: χ^2^, *df*, *p* value		3.54, 2, 0.171		26.69, 2, <0.001		9.160, 2, 0.010

*Tables 2 and 3: Age of arrival was categorised in these broad age groups. The matching of cases to controls was done in a more fine-grained way by categorising calendar year of birth in 5-year groups (see Methods).

For those from other non-Western countries, the IRRs were higher than 1.00 in all age groups already 2–3 years after arrival (Table 3); after exclusion of prevalent cases among controls, the IRRs were already significantly higher than 1.00 in all age groups in the year of arrival, and without a trend to higher values thereafter (online Supplementary Table S7).

For migrants from Eastern Europe, similar results were found for those aged 20–40 and 40–60 years (Table 3), and higher values for the IRR in an earlier phase when excluding prevalent cases among controls (online Supplementary Table S7). For other Western migrants, lower IRRs were found in all age groups and all years after arrival, and no IRRs significantly higher than 1.00 could be established, irrespective of in- or exclusion of prevalent cases in the reference population (Table 3 and online Supplementary Table S7).

## Discussion

This cohort study assessed the course of the relative risk of a psychotic disorder among migrants after their arrival in the Netherlands. In general, the IRRs for Dispensings and Treatments were lower than 1.00 shortly after arrival and increased to values significantly higher than one during the years thereafter. For Treatments, however, IRRs higher than 1.00 were already found from the second year after arrival onwards, especially for migrants from non-Western countries. Furthermore, among migrants aged 10–20 years at arrival, and among migrants from sub-Saharan Africa, IRRs of Treatments significantly higher than 1.00 were already found for the year of arrival.

Our sensitivity analysis showed that the low IRRs of Treatment in the year of arrival may be due to a relatively high number of prevalent cases in the reference population and the increasing trend therafter due to the fast ‘fading away’ of these cases after being established as incident (a person with incident psychosis within the time window of observation is excluded from the analysis after the calendar year of diagnosis). These findings do not contradict our hypothesis of a HME for the majority of migrants, when this effect is understood as a comparatively low prevalence at the time of arrival. However, they challenge our hypothesis of a HME, when it is operationalised as a comparatively low incidence in the early years after arrival and a gradually increasing relative risk due to long-term effects of social stressors in the host country. The results suggest that, indeed, the prevalence of psychosis among migrants is, in general, comparatively low at arrival and that the increase of the IRR occurs early and remains relatively stable within the time window of our study.

### Strengths and limitations

This is the first study to examine the time course of the relative risk of psychosis for migrants.

Previous studies that compared the risk of psychosis among migrants to that for the native-born population did not consider the time interval since arrival, while this is important for our understanding of the aetiology of the increased risk for migrants (Selten et al., 2020; Termorshuizen et al., 2020). We used a large longitudinal population-based register with well-defined and well-registered data on dates of birth, death, immigration and emigration, country(ies) of origin of the individual and his/ her parents. The study had a number of potential limitations.

First, APM is often prescribed for non-psychotic disorders. For this reason we also used a registered diagnosis of psychosis from insurance claims for mental health care as outcome. An analysis of the relationship between these two outcomes indicated that Dispensing as a proxy for psychosis may underestimate the true relative risk, even though this proxy outcome can be used to follow trends. The low IRRs of Dispensing in the early years after arrival do not necessarily point to ascertainment bias, but may also be explained by higher levels of off-label use among native-born controls (Boonstra, Grobbee, Hak, Kahn, & Burger, 2011). The lower IRRs of Dispensing compared to the IRRs of Treatment in the early years after arrival and the increasing trend towards higher values thereafter may indicate a shift in the utilisation of medication towards the levels among natives.

Secondly, the follow-up started in the calendar year of arrival, disregarding previous information for both migrants and their matched controls. Thus, an incident case within the time window of observation may actually be a prevalent case when seen from a lifetime perspective. A second analysis that excluded native-born controls with a dispensed APM and/or a DTC in the year prior to arrival of the matched migrant indeed showed that the high rates of psychosis among native-born controls reflect many lifetime-prevalent cases. Obviously, the IRRs during the early years after arrival do not reflect ratios of lifetime-incidence figures. We probably compared two populations with large differences in lifetime-prevalence rates. On the other hand, the low IRRs in the year of arrival and the early years thereafter yielded by the first analysis point to the HME (if one defines this as a low prevalence, see above) that we wished to demonstrate. In the later years after arrival, the IRRs are increasingly likely to reflect the ratio of lifetime-incidence figures.

As no information on health-care utilisation before immigration was available, we could not exclude prevalent cases among migrants. Consequently, we regarded the analysis after exclusion of native-born controls with a registered diagnosis prior to the date of arrival of their matched migrants as secondary to assess the potential influence of prevalent cases among the native-born controls on the results. The big advantage of our primary analysis is that by disregarding information on health care utilisation for native-born controls prior to arrival, migrants and controls were treated alike. The interpretation of the results is not so simple, as the distinction between incidence and prevalence is ignored, but the comparison of the rates of psychosis, with identical outcome definitions for both groups, itself is valid. One could suggest that the low psychosis rates for migrants in the year of arrival in the first analysis do not reflect a true rate, but a high threshold for treatment. A first-contact incidence study in The Netherlands, however, showed that most migrants from non-western countries reached the services within a year (Selten et al., 2001). A systematic review did not find racial or ethnic differences in the duration of untreated psychosis (Anderson, Flora, Archie, Morgan, & McKenzie, 2014). Thus, the low rates in the year of arrival may reflect a comparatively low prevalence among migrants and thus a real HME.

It is true, we cannot definitively rule out a delay in the onset of treatment as an alternative explanation for the lower risk in the year of arrival. Notably, previous studies from the Netherlands did not provide information on the duration of untreated psychosis among migrants from Western countries. However, if higher treatment thresholds and ascertainment bias play an important role, our final conclusion of a relative risk significantly higher than 1.00 in the early years after arrival becomes stronger, not weaker.

Thirdly, Dispensings and Treatments were analysed as dichotomous variables for a calendar year, disregarding the precise dates of starting and stopping. The time in the denominator was assessed in a more fine-grained way as the number of days of follow-up for each calendar year, using the precise dates of birth and death, immigration and emigration and was truncated in the calendar year of incident psychosis. Thus, the ratio of outcome to time, albeit having some random error, was reasonably well defined, and no systematic over- or underestimation favouring migrants or native-born controls is to be expected. There is a risk of underestimation of the RR for Dispensings if migrants more often use APM during an in-patient treatment of a whole calendar year. However, such treatments are rare.

Fourth, no data were available to examine insurance claims for psychosis over a period longer than 6 years.

### Interpretation of findings

How should we interpret the high relative risks in an early stage for migrants from non-Western countries, especially sub-Saharan Africa, and Eastern Europe? Migrants from Eastern Europe, mostly from Poland, the former Soviet Union and Bulgaria, usually perform low-skilled work in the agricultural sector, while those from Western Europe are often highly educated knowledge workers (de Boom, Weltevrede, Rezai, & Engbersen, 2008). Since migrants from Eastern Europe and non-Western countries share a low income and status, our findings are compatible with the social defeat hypothesis of psychosis, which posits that the chronic experience of a subordinate or outsider position increases the risk, possibly by sensitising a dopamine pathway (Egerton et al., 2017; McCutcheon, Abi-Dargham, & Howes, 2019; Selten & Cantor-Graae, 2005). Other related stressors proposed to contribute to the increased risk among migrants include the stress of acculturation, linguistic distance and discrimination (Dykxhoorn & Kirkbride, 2019; Jongsma et al., 2021; Stilo et al., 2017). However, since the effect of social stressors is supposed to become more serious with lapse of time, it is uncertain whether they fully explain the high relative risks already in the early years following arrival. It is also possible that stressors present in the country of origin or during the process of migration contribute to these relative risks. Furthermore, our findings do not exclude an important role of social stressors in the host country at time intervals beyond those of the present study.

The IRRs for migrants from sub-Saharan Africa, especially at a young age, were strikingly high in the year of arrival, even if all prevalent cases of psychosis among controls were included. Since many of them are survivors of a difficult refugee process, traumatic experiences in the region of origin and/ or during the flight may play an important role. A case-control study of migrants reported that not only social disadvantages after migration, but also stressors before or during migration were associated with an increased risk of psychosis (Tarricone et al., 2021). This explanation is supported by a Swedish study reporting an increased risk of psychosis among refugees compared to non-refugee migrants [HR = 1.7 (1.3–2.1)] and a shorter median duration between arrival and diagnosis (2.8 *v.* 3.9 years) (Hollander et al., 2016). Our findings also raise questions about the prevalence of psychosis in Africa and underline the importance of conducting epidemiological studies in this continent.

Since the HME may not apply to all groups of migrants, this phenomenon should be approached in a more differentiated way. The HME may not be equally valid for all categories of migrants. Our results agree nicely with the findings by Helgesson et al., who reported a HME for a group of mainly labour migrants (Western), but not for a group of mainly refugee and family reunion migrants (non-Western) (Helgesson, Johansson, Nordquist, Vingard, & Svartengren, 2019). Further studies using in-depth information on socio-economic position, reasons for departure and experiences before, during and after migration may elucidate the aetiology of the differences in results.

## Conclusions

This study of migrants found evidence of a comparatively low prevalence of treatment for psychotic disorder in the year of arrival and of a high incidence already in the early years thereafter. The figures differed markedly across regions of origin. Young migrants from sub-Saharan Africa were already at a very high risk in the year of their arrival. Those who are responsible for the integration of migrants (e.g. politicians, staff at asylum centres) should be aware of their high risk of mental health problems shortly after arrival.

## Supplementary material

For supplementary material accompanying this paper visit https://doi.org/10.1017/S0033291722001192. click here to view supplementary material
