# Supplementary material for: Risk of psychosis among migrants to the Netherlands by time since arrival
Source: Psychol Med. 2022 May 5;53(10):4395–404. doi: 10.1017/S0033291722001192 (PMC10388323; doi:10.1017/S0033291722001192)
Supplement: Supplementary file 1 [file S0033291722001192sup001.docx]

Supplemental Results

*Association between Dispensing and Treatment*

Supplemental Table 8 shows the proportions of persons with Treatment among those with and among those without Dispensing for the years 2011-2016, including all native-born persons of the reference population. There were few persons with Treatment among those without Dispensing, as expected. On the other hand, among those with Dispensing many had no associated Treatment. Especially in the later years after arrival, the native-born controls often have lower proportions of Treatment among those with Dispensing, which suggests more off-label prescriptions of AP among native-born controls compared to migrants. Native-born controls also have lower percentages of Treatment among those without Dispensing at later years. These figures were used to reclassify the numbers with and without Dispensing, and to recalculate the estimated IRRs of Dispensing accordingly (last column). After recalculation, the IRRs became higher and higher at earlier years following arrival. In Supplemental Tables 9.A-9.D, the association between Dispensing and Treatment and recalculated IRRs of Dispensing are shown for the separate migrant groups.

Supplemental Figure

Supplemental Figure 1. Migrants to the Netherlands vs. native-born controls (=Reference population): Incidence Rate Ratio (IRR) of Incident Diagnosis Treatment Combinations for psychosis (IDTCs, 2011-2016), by number of years since arrival. The dotted line shows the results after exclusion of controls with a registered DTC for psychosis in the year before the arrival of their matched migrant (‘prevalent cases’).


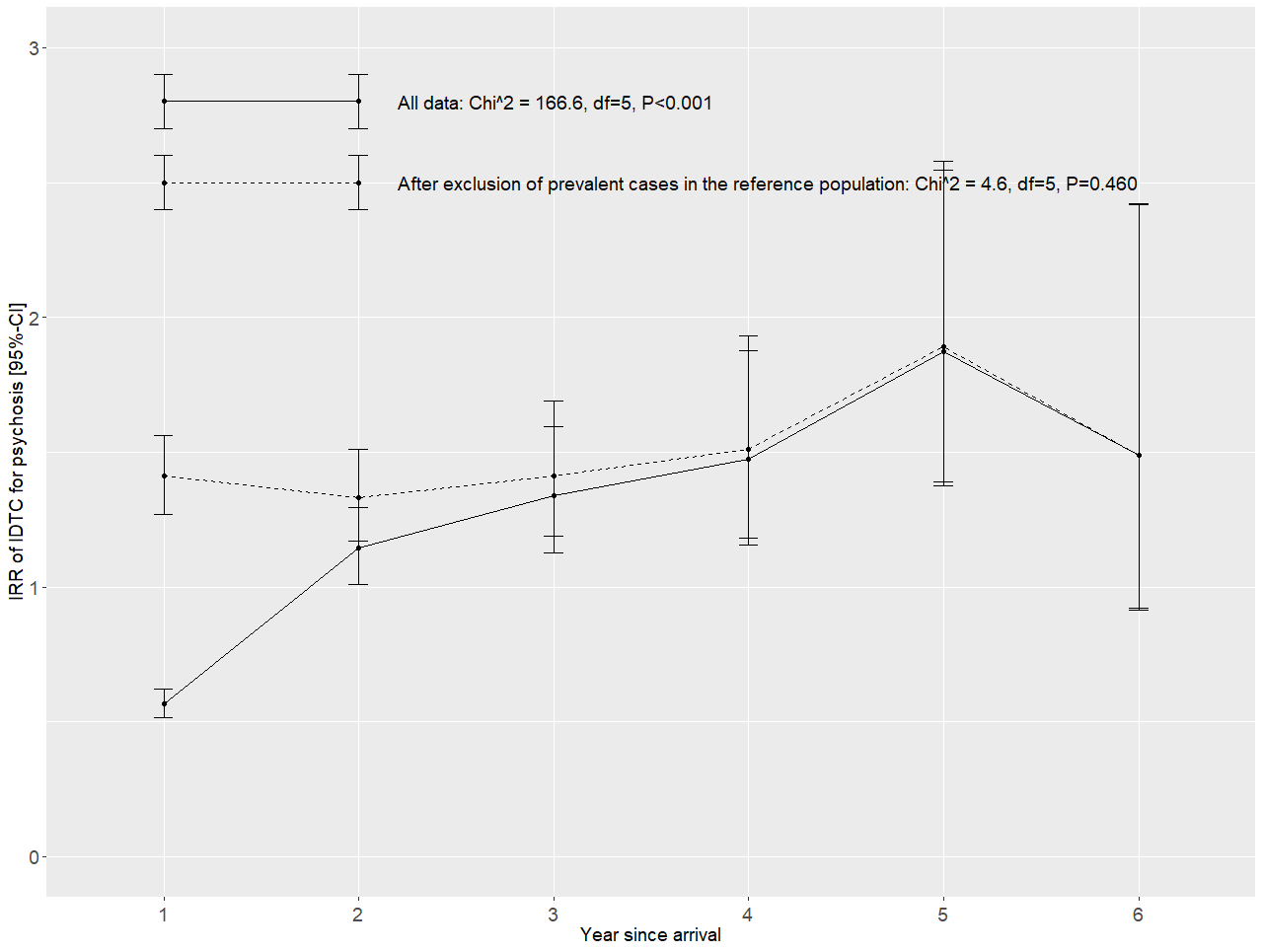


Supplemental Tables

Supplemental Table 1. Demographic data on migrants to the Netherlands, arrived within the period 2006-2017, and their matched native-born controls (=Reference population).

|  | Migrants |  |  | Reference population |  |
| --- | --- | --- | --- | --- | --- |
|  | N | % |  | N | % |
| Total | 1,281,678 | 100% |  | 2,542,313 | 100% |
|  |  |  |  |  |  |
| Numbers of controls |  |  |  |  |  |
| 1 unique control |  |  |  | 21,043 | 0.8% |
| Nr. 1 of the two controls |  |  |  | 1,260,635 | 49.6% |
| Nr. 2 of the two controls |  |  |  | 1,260,635 | 49.6% |
|  |  |  |  |  |  |
| Gender Male | 650,436 | 50.8% |  | 1,292,259 | 50.7% |
| Female | 631,242 | 49.2% |  | 1,250,054 | 49.3% |
|  |  |  |  |  |  |
| Age at arrival 10-15 yr | 47,408 | 3.7% |  | 94,808 | 3.7% |
| 15-20 yr | 122,403 | 9.6% |  | 244,289 | 9.6% |
| 20-40 yr | 939,307 | 73.3% |  | 1,858,177 | 73.1% |
| 40-60 yr | 157,124 | 12.3% |  | 314,237 | 12.4% |
| >60 yr | 15,436 | 1.2% |  | 30,802 | 1.2% |
|  |  |  |  |  |  |
| Region of origin of migrant |  |  |  |  |  |
| Non-Western |  |  |  |  |  |
| Sub-Saharan Africa | 87,450 | 6.8% |  | 173,649 | 6.8% |
| Other non-Western | 449,430 | 35.1% |  | 891,501 | 35.1% |
|  |  |  |  |  |  |
| Western |  |  |  |  |  |
| Eastern Europe (including FSU) | 367,011 | 28.6% |  | 727,637 | 28.6% |
| Other Western | 377,670 | 29.5% |  | 749,292 | 29.5% |
|  |  |  |  |  |  |
| Other/ unknown | 117 | 0.009% |  | 234 | 0.009% |
|  |  |  |  |  |  |
| Calendar year of arrival |  |  |  |  |  |
| 2006 | 60,563 | 4.7% |  | 120,902 | 4.8% |
| 2007 | 72,234 | 5.6% |  | 144,000 | 5.7% |
| 2008 | 93,144 | 7.3% |  | 185,356 | 7.3% |
| 2009 | 93,635 | 7.3% |  | 186,090 | 7.3% |
| 2010 | 96,584 | 7.5% |  | 191,677 | 7.5% |
| 2011 | 103,609 | 8.1% |  | 205,389 | 8.1% |
| 2012 | 99,865 | 7.8% |  | 197,873 | 7.8% |
| 2013 | 103,884 | 8.1% |  | 205,761 | 8.1% |
| 2014 | 117,978 | 9.2% |  | 233,630 | 9.2% |
| 2015 | 133,092 | 10.4% |  | 263,486 | 10.4% |
| 2016 | 153,416 | 12.0% |  | 303,864 | 12.0% |
| 2017 | 153,674 | 12.0% |  | 304,285 | 12.0% |
|  |  |  |  |  |  |

Supplemental Table 2. Demographic data on migrants to the Netherlands, arrived within the period 2011-2016, and their matched native-born controls (=Reference population).

|  | Migrants |  |  | Reference population |  |
| --- | --- | --- | --- | --- | --- |
|  | N | % |  | N | % |
| Total | 711,844 |  |  | 1,410,003 |  |
|  |  |  |  |  |  |
| Numbers of controls |  |  |  |  |  |
| 1 unique control | 711,844 | 100.0% |  | 13,685 | 1.0% |
| Nr. 1 of the two controls | 0 | 0.0% |  | 698,159 | 49.5% |
| Nr. 2 Of the two controls | 0 | 0.0% |  | 698,159 | 49.5% |
|  |  |  |  |  |  |
| Gender Male | 362,649 | 50.9% |  | 719,535 | 51.0% |
| Female | 349,195 | 49.1% |  | 690,468 | 49.0% |
|  |  |  |  |  |  |
| Age at arrival 10-15 yr | 26,119 | 3.7% |  | 52,238 | 3.7% |
| 15-20 yr | 71,558 | 10.1% |  | 143,033 | 10.1% |
| 20-40 yr | 519,935 | 73.0% |  | 1,026,310 | 72.8% |
| 40-60 yr | 85,788 | 12.1% |  | 171,571 | 12.2% |
| >60 yr | 8,444 | 1.2% |  | 16,851 | 1.2% |
|  |  |  |  |  |  |
| Region of origin of migrant |  |  |  |  |  |
| Non-Western |  |  |  |  |  |
| Sub-Saharan Africa | 44,985 | 6.3% |  | 89,165 | 6.3% |
| Other non-Western | 250,023 | 35.1% |  | 495,241 | 35.1% |
|  |  |  |  |  |  |
| Western |  |  |  |  |  |
| Eastern Europe (including FSU) | 208,172 | 29.2% |  | 412,041 | 29.2% |
| Other Western | 208,585 | 29.3% |  | 413,398 | 29.3% |
|  |  |  |  |  |  |
| Other/ unknown | 79 | 0.01% |  | 158 | 0.01% |
|  |  |  |  |  |  |
| Calendar Year of arrival |  |  |  |  |  |
| 2011 | 103,609 | 14.6% |  | 205,389 | 14.6% |
| 2012 | 99,865 | 14.0% |  | 197,873 | 14.0% |
| 2013 | 103,884 | 14.6% |  | 205,761 | 14.6% |
| 2014 | 117,978 | 16.6% |  | 233,630 | 16.6% |
| 2015 | 133,092 | 18.7% |  | 263,486 | 18.7% |
| 2016 | 153,416 | 21.6% |  | 303,864 | 21.6% |
|  |  |  |  |  |  |

Supplemental Table 3. (see Table 1.) Migrants to the Netherlands vs. native-born controls (=Reference population): numbers of persons, numbers of Incident Dispensings of Antipsychotic Medication (IDAPs, 2006-2017) and of Incident Diagnosis Treatment Combinations for psychosis (IDTCs, 2011-2016), Rates (number/ 10,000 person-years), and Incidence Rate Ratios (IRRs), after exclusion of controls with a registered AP or a registered DTC for psychosis, respectively, in the year before the arrival of their matched migrant (‘prevalent cases’), by number of years since arrival.

|  | Reference population  After exclusion of prevalent cases | | |  | Migrants |  |  |  |
| --- | --- | --- | --- | --- | --- | --- | --- | --- |
| Years since arrival | N persons | Number of IDAPs/ IDCTs | Rate (/10,000 person-years |  | N persons | Number of IDAPs/ IDCTs | Rate (/10,000 person-years) | IRR [95%-CI] |
|  |  |  |  |  |  |  |  |  |
| IDAP |  |  |  |  |  |  |  |  |
| 1 | 2,525,038 | 5,957 | 23.6 |  | 1,281,678 | 955 | 16.6 | 0.70 [0.66-0.75] |
| 2 | 2,010,661 | 4,029 | 20.1 |  | 1,021,490 | 1,324 | 14.7 | 0.73 [0.69-0.78] |
| 3 | 1,331,311 | 2,469 | 18.6 |  | 676,118 | 972 | 15.7 | 0.85 [0.79-0.91] |
| 4 | 938,406 | 1,691 | 18.1 |  | 476,349 | 766 | 17.3 | 0.96 [0.88-1.04] |
| 5 | 691,600 | 1,215 | 17.6 |  | 350,643 | 568 | 17.1 | 0.97 [0.88-1.07] |
| 6 | 524,994 | 943 | 18.0 |  | 265,881 | 504 | 19.8 | 1.10 [0.99-1.23] |
| 7 | 403,733 | 711 | 17.6 |  | 204,138 | 377 | 19.2 | 1.09 [0.96-1.23] |
| 8 | 299,519 | 501 | 16.8 |  | 151,176 | 297 | 20.3 | 1.21 [1.05-1.40] |
| 9 | 214,828 | 370 | 17.3 |  | 108,209 | 219 | 20.8 | 1.20 [1.02-1.42] |
| >=10 | 257,700 | 387 | 15.0 |  | 129,423 | 266 | 21.0 | 1.39 [1.19-1.63] |
|  |  |  |  |  |  | Year 1-10: χ2, df, P value | | 178.3, 9, <0.001 |
| IDTC |  |  |  |  |  |  |  |  |
| 1 | 1,406,794 | 1,501 | 10.7 |  | 711,844 | 477 | 15.0 | 1.41 [1.27-1.56] |
| 2 | 988,104 | 633 | 6.4 |  | 500,001 | 373 | 8.5 | 1.33 [1.17-1.51] |
| 3 | 561,989 | 316 | 5.6 |  | 284,196 | 206 | 8.0 | 1.41 [1.19-1.69] |
| 4 | 329,151 | 156 | 4.7 |  | 166,328 | 111 | 7.2 | 1.51 [1.18-1.93] |
| 5 | 185,468 | 85 | 4.6 |  | 93,637 | 77 | 8.7 | 1.89 [1.39-2.58] |
| 6 | 8,5351 | 39 | 4.6 |  | 43,011 | 28 | 6.8 | 1.49 [0.92-2.42] |
|  |  |  |  |  |  | Year 1-6: χ2, df, P value | | 4.6, 5, 0.460 |

Supplemental Table 4. (see Figure 2.) Migrants to the Netherlands vs. native-born controls (=Reference population): numbers of persons, numbers of Incident Diagnosis Treatment Combinations for psychosis (IDTCs, 2011-2016), Rates (number/ 10,000 person-years), and Incidence Rate Ratios (IRRs), by number of years since arrival and region of origin.

|  | Reference population  All data | |  |  | Migrants |  |  |  |
| --- | --- | --- | --- | --- | --- | --- | --- | --- |
| Region of origin  Years since arrival | N persons | Number of IDTCs | Rate (/10,000 person-years) |  | N persons | Number of IDTCs | Rate (/10,000 person-years) | IRR [95%-CI] |
| Non-Western: 1 | 89,165 | 191 | 21.4 |  | 44,985 | 111 | 52.7 | 2.46 [1.95-3.11] |
| sub-Saharan Africa 2 | 66,246 | 39 | 5.9 |  | 33,396 | 67 | 21.3 | 3.62 [2.44-5.37] |
| 3 | 40,583 | 24 | 5.9 |  | 20,397 | 37 | 19.3 | 3.25 [1.95-5.44] |
| 4-6 | 44,561 | 21 | 4.7 |  | 22,283 | 36 | 16.7 | 3.54 [2.07-6.06] |
|  |  |  |  |  |  | Year 1-6: χ2, df, P value | | 3.79, 3, 0.285 |
| Non-Western: Other 1 | 495,241 | 1,261 | 25.5 |  | 250,023 | 169 | 14.7 | 0.58 [0.49-0.68] |
| 2 | 340,487 | 223 | 6.6 |  | 172,176 | 131 | 8.4 | 1.28 [1.03-1.59] |
| 3 | 197,083 | 123 | 6.3 |  | 99,598 | 90 | 9.7 | 1.55 [1.18-2.04] |
| 4 | 119,375 | 66 | 5.5 |  | 60,290 | 36 | 6.3 | 1.14 [0.76-1.70] |
| 5 | 70,482 | 32 | 4.5 |  | 35,590 | 35 | 10.2 | 2.25 [1.39-3.64] |
| 6 | 33,953 | 15 | 4.4 |  | 17,113 | 13 | 7.9 | 1.78 [0.85-3.74] |
|  |  |  |  |  |  | Year 1-6: χ2, df, P value | | 72.84, 5, <0.001 |
| Western: 1 | 412,041 | 1,195 | 29.0 |  | 208,172 | 132 | 14.3 | 0.49 [0.41-0.59] |
| Eastern-European 2 | 289,320 | 231 | 8.0 |  | 146,442 | 109 | 8.7 | 1.09 [0.87-1.36] |
| 3 | 167,054 | 100 | 6.0 |  | 84,567 | 50 | 6.5 | 1.08 [0.77-1.52] |
| 4 | 99,663 | 51 | 5.1 |  | 50,443 | 39 | 8.3 | 1.61 [1.06-2.44] |
| 5-6 | 83,878 | 40 | 4.8 |  | 42,364 | 29 | 7.2 | 1.50 [0.93-2.42] |
|  |  |  |  |  |  | Year 1-6: χ2, df, P value | | 55.7, 4, P<0.001 |
| Western: Other 1 | 413,398 | 1,086 | 26.3 |  | 208,585 | 65 | 7.3 | 0.28 [0.22-0.36] |
| 2 | 292,549 | 244 | 8.4 |  | 147,941 | 66 | 5.3 | 0.64 [0.48-0.83] |
| 3 | 157,513 | 87 | 5.5 |  | 79,620 | 29 | 4.2 | 0.75 [0.49-1.14] |
| 4 | 86,673 | 35 | 4.0 |  | 43,793 | 14 | 3.6 | 0.89 [0.48-1.65] |
| 5-6 | 61,543 | 25 | 4.1 |  | 31,092 | 14 | 4.9 | 1.21 [0.63-2.34] |
|  |  |  |  |  |  | Year 1-6: χ2, df, P value | | 38.4, 4, <0.001 |

Supplemental Table 5. Migrants to the Netherlands vs. native-born controls (=Reference population): numbers of persons, numbers of Incident Diagnosis Treatment Combinations for psychosis (IDTCs, 2011-2016), Rates (number/ 10,000 person-years), and Incidence Rate Ratios (IRRs), after exclusion of controls with a registered DTC for psychosis in the year before the arrival of their matched migrant (‘prevalent cases’), by number of years since arrival and region of origin.

|  | Reference population  After exclusion of prevalent cases | | |  | Migrants |  |  |  |
| --- | --- | --- | --- | --- | --- | --- | --- | --- |
| Region of origin  Years since arrival | N persons | Number of IDTCs | Rate (/10,000 person-years) |  | N persons | Number of IDTCs | Rate (/10,000 person-years) | IRR [95%-CI] |
| Non-Western: 1 | 89,013 | 91 | 10.2 |  | 44,985 | 111 | 52.7 | 5.16 [3.91-6.80] |
| sub-Saharan Africa 2 | 66,230 | 34 | 5.1 |  | 33,396 | 67 | 21.3 | 4.15 [2.75-6.27] |
| 3 | 40,565 | 23 | 5.7 |  | 20,397 | 37 | 19.3 | 3.39 [2.02-5.71] |
| 4-6 | 44,552 | 21 | 4.7 |  | 22,283 | 36 | 16.7 | 3.54 [2.06-6.06] |
|  |  |  |  |  |  | Year 1-6: χ2, df, P value | | 2.93, 3, 0.403 |
| Non-Western: Other 1 | 494,095 | 452 | 9.1 |  | 250,023 | 169 | 14.7 | 1.61 [1.35-1.92] |
| 2 | 340,294 | 193 | 5.7 |  | 172,176 | 131 | 8.4 | 1.48 [1.19-1.85] |
| 3 | 196,991 | 117 | 6.0 |  | 99,598 | 90 | 9.7 | 1.63 [1.24-2.15] |
| 4 | 119,327 | 66 | 5.5 |  | 60,290 | 36 | 6.3 | 1.14 [0.76-1.70] |
| 5 | 70,462 | 32 | 4.5 |  | 35,590 | 35 | 10.2 | 2.25 [1.39-3.64] |
| 6 | 33,953 | 15 | 4.4 |  | 17,113 | 13 | 7.9 | 1.78 [0.85-3.74] |
|  |  |  |  |  |  | Year 1-6: χ2, df, P value | | 5.13, 5, 0.400 |
| Western: 1 | 411,046 | 493 | 12.0 |  | 208,172 | 132 | 14.3 | 1.19 [0.98-1.44] |
| Eastern-European 2 | 289,135 | 194 | 6.7 |  | 146,442 | 109 | 8.7 | 1.29 [1.02-1.63] |
| 3 | 166,966 | 93 | 5.6 |  | 84,567 | 50 | 6.5 | 1.16 [0.83-1.64] |
| 4 | 99,632 | 49 | 4.9 |  | 50,443 | 39 | 8.3 | 1.68 [1.10-2.55] |
| 5-6 | 83,863 | 39 | 4.7 |  | 42,364 | 29 | 7.2 | 1.54 [0.95-2.49] |
|  |  |  |  |  |  | Year 1-6: χ2, df, P value | | 3.00, 4, 0.558 |
| Western: Other 1 | 412,483 | 465 | 11.3 |  | 208,585 | 65 | 7.3 | 0.65 [0.50-0.84] |
| 2 | 292,352 | 212 | 7.3 |  | 147,941 | 66 | 5.3 | 0.73 [0.55-0.96] |
| 3 | 157,439 | 83 | 5.3 |  | 79,620 | 29 | 4.2 | 0.79 [0.52-1.20] |
| 4 | 86,638 | 33 | 3.8 |  | 43,793 | 14 | 3.6 | 0.94 [0.50-1.76] |
| 5-6 | 61,527 | 25 | 4.1 |  | 31,092 | 14 | 4.9 | 1.21 [0.63-2.33] |
|  |  |  |  |  |  | Year 1-6: χ2, df, P value | | 3.94, 4, 0.415 |

Suppl Table 6. Migrants to the Netherlands vs. native-born controls (=Reference population): see Suppl Table 3., by number of years since arrival and age.

|  | Reference population  After exclusion of prevalent cases | | |  | Migrants |  |  |  |
| --- | --- | --- | --- | --- | --- | --- | --- | --- |
| Age at arrival  Years since arrival | N persons | Number of IDAPs/ IDTCs | Rate (/10,000 person-years) |  | N persons | Number of IDAPs/ IDTCs | Rate (/10,000 person-years) | IRR [95%-CI] |
| IDAP 10-20 years 1 | 336,872 | 752 | 22.3 |  | 169,811 | 106 | 14.7 | 0.66 [0.54-0.81] |
| 2-3 | 463,637 | 866 | 18.7 |  | 234,126 | 278 | 12.8 | 0.68 [0.60-0.78] |
| 4-6 | 314,028 | 588 | 18.8 |  | 158,718 | 225 | 15.0 | 0.80 [0.69-0.93] |
| >6 | 165,562 | 321 | 19.4 |  | 83,550 | 189 | 23.4 | 1.20 [1.01-1.44] |
|  |  |  |  |  |  | Year 1-10: χ2, df, P value | | 28.7, 3, <0.001 |
| 20-40 years 1 | 1,846,310 | 4,249 | 23.0 |  | 939,307 | 549 | 13.0 | 0.56 [0.52-0.62] |
| 2-3 | 2,416,721 | 4,590 | 19.0 |  | 1,230,485 | 1,483 | 13.6 | 0.71 [0.67-0.76] |
| 4-6 | 1,528,356 | 2,565 | 16.8 |  | 776,846 | 1,206 | 16.5 | 0.98 [0.92-1.05] |
| >6 | 839,013 | 1,267 | 15.1 |  | 423,700 | 744 | 18.1 | 1.20 [1.09-1.31] |
|  |  |  |  |  |  | Year 1-10: χ2, df, P value | | 182.3, 3, <0.001 |
| 40-60 years 1 | 311,378 | 807 | 25.9 |  | 157,124 | 188 | 25.3 | 0.97 [0.83-1.14] |
| 2-3 | 421,167 | 808 | 19.2 |  | 212,614 | 400 | 20.6 | 1.07 [0.95-1.21] |
| 4-6 | 285,264 | 513 | 18.0 |  | 143,615 | 327 | 24.0 | 1.33 [1.16-1.53] |
| >6 | 157,822 | 266 | 16.9 |  | 78,982 | 181 | 23.5 | 1.39 [1.15-1.68] |
|  |  |  |  |  |  | Year 1-10: χ2, df, P value | | 13.8, 3, 0.0032 |
| IDTC 10-20 years 1 | 195,180 | 75 | 3.8 |  | 97,677 | 43 | 10.6 | 2.75 [1.89-4.00] |
| 2-3 | 222,344 | 95 | 4.3 |  | 11,1240 | 68 | 6.6 | 1.54 [1.13-2.11] |
| 4-6 | 90,530 | 44 | 4.9 |  | 45,258 | 35 | 8.2 | 1.69 [1.09-2.64] |
|  |  |  |  |  |  | Year 1-6: χ2, df, P value | | 5.7, 2, 0.058 |
| 20-40 years 1 | 1,023,864 | 1,190 | 11.6 |  | 519,935 | 342 | 14.7 | 1.26 [1.12-1.43] |
| 2-3 | 1,116,016 | 739 | 6.6 |  | 566,750 | 412 | 8.3 | 1.25 [1.11-1.41] |
| 4-6 | 421,723 | 207 | 4.9 |  | 213,818 | 143 | 7.1 | 1.45 [1.17-1.80] |
|  |  |  |  |  |  | Year 1-6: χ2, df, P value | | 1.5, 2, 0.473 |
| 40-60 years 1 | 170,940 | 215 | 12.6 |  | 85,788 | 83 | 20.4 | 1.62 [1.26-2.09] |
| 2-3 | 193,095 | 108 | 5.6 |  | 96,854 | 91 | 10.3 | 1.85 [1.40-2.44] |
| 4-6 | 79,904 | 25 | 3.1 |  | 39,991 | 33 | 8.7 | 2.78 [1.65-4.67] |
|  |  |  |  |  |  | Year 1-6: χ2, df, P value | | 3.37, 2, 0.186 |

Supplemental Table 7. Migrants to the Netherlands vs. native-born controls (=Reference population): Rates for migrants (number/ 10,000 person-years) and Incidence Rate Ratios (IRRs) of Incident Diagnosis Treatment Combinations (IDTCs, 2011-2016), after exclusion of controls with a registered DTC for psychosis in the year before the arrival of their matched migrant (‘prevalent cases’), by number of years since arrival, age, and region of origin.

| Age at arrival | 10-20 year |  | 20-40 year |  | 40-60 year |  |
| --- | --- | --- | --- | --- | --- | --- |
|  | Rate (/10,000 person-years) | IRR [95%-CI] | Rate (/10,000 person-years) | IRR [95%-CI] | Rate (/10,000 person-years) | IRR [95%-CI] |
| Years since arrival |  |  |  |  |  |  |
|  |  |  |  |  |  |  |
| Non-Western migrants: other 1 | 9.9 | 2.74 [1.50-5.01] | 13.7 | 1.35 [1.09-1.67] | 25.2 | 2.38 [1.59-3.56] |
| 2-3 | 7.4 | 2.18 [1.30-3.66] | 8.4 | 1.26 [1.03-1.55] | 14.5 | 3.60 [2.22-5.84] |
| 4-6 | 8.0 | 1.45 [0.73-2.87] | 6.9 | 1.34 [0.95-1.91] | 10.7 | 2.91 [1.29-6.56] |
| Year 1-6: χ2, df, P value |  | 1.90, 2, 0.387 |  | 0.23, 2, 0.891 |  | 1.66, 2, 0.436 |
|  |  |  |  |  |  |  |
| Non-Western migrants: Sub-Saharan Africa 1 | 32.9 | 7.31 [3.37-15.83] | 62.4 | 4.96 [3.61-6.82] | 48.9 | 5.81[2.34-14.44] |
| 2-3 | 17.8 | 8.27 [3.38- 20.22] | 23.4 | 3.44 [2.37-4.98] | 13.0 | 2.50 [0.84-7.44] |
| 4-6 | 16.8 | 10.25[2.25-46.79] | 19.0 | 2.92 [1.57-5.43] | 7.4 | 2.08 [0.29-14.73] |
| Year 1-6: χ2, df, P value |  | 0.16, 2, 0.923 |  | 3.39, 2, 0.183 |  | 1.77, 2, 0.414 |
|  |  |  |  |  |  |  |
| Western migrants: Eastern Europe 1 | 8.7 | 2.62 [1.06-6.50] | 14.3 | 1.13 [0.91-1.40] | 16.9 | 1.12 [0.70-1.80] |
| 2-3 | 3.2 | 0.65 [0.28-1.50] | 8.3 | 1.29 [1.03-1.60] | 8.7 | 1.26 [0.76-2.07] |
| 4-6 | 6.3 | 1.16 [0.43-3.14] | 7.5 | 1.48 [1.02-2.13] | 10.5 | 3.88 [1.55-9.72] |
| Year 1-6: χ2, df, P value |  | 4.92, 2, 0.085 |  | 1.73, 2, 0.421 |  | 5.73, 2, 0.057 |
|  |  |  |  |  |  |  |
| Western migrants: other 1 | 1.0 | 0.23 [0.03-1.72] | 7.1 | 0.59 [0.43-0.79] | 12.1 | 0.93 [0.51-1.69] |
| 2-3 | 2.8 | 0.48 [0.22-1.04] | 4.9 | 0.72 [0.55-0.94] | 6.5 | 1.07 [0.59-1.95] |
| 4-6 | 5.3 | 1.03 [0.36-2.98] | 3.8 | 0.96 [0.55-1.67] | 4.1 | 1.45 [0.41-5.12] |
| Year 1-6: χ2, df, P value |  | 2.18, 2, 0.337 |  | 2.58, 2, 0.275 |  | 0.42, 2, 0.812 |
|  |  |  |  |  |  |  |

Supplemental Table 8. Proportions of Incident Diagnosis Treatment Combination (IDTC, 2011-2016) among those with and among those without Incident Dispensing of Antipsychotic medication (IDAP, 2011-2016), for migrants (all categories for country of origin) and for native-born controls, by number of years since and age at arrival. The IRR of IDAP was re-estimated using these percentages.

|  | % IDTC among those with IDAP, native-born controls | % IDTC among those without IDAP, native-born controls |  | % IDTC among those with IDAP, migrants | % IDTC among those without IDAP, migrants |  | IRR of IDAP  Original | IRR of IDAP  Re-estimated |
| --- | --- | --- | --- | --- | --- | --- | --- | --- |
| All ages |  |  |  |  |  |  |  |  |
| Years since arrival 1 | 26.7% | 0.063% |  | 29.1% | 0.046% |  | 0.22 | 0.58 |
| 2-3 | 12.3% | 0.021% |  | 23.2% | 0.040% |  | 0.72 | 1.68 |
| 4-6 | 10.5% | 0.014% |  | 22.2% | 0.031% |  | 0.97 | 2.17 |
|  |  |  |  |  |  |  |  |  |
| Age at arrival |  |  |  |  |  |  |  |  |
| 1. 10-20 year |  |  |  |  |  |  |  |  |
| 1 | 5.8% | 0.023% |  | 22.4% | 0.031% |  | 0.21 | 1.70 |
| 2-3 | 9.9% | 0.015% |  | 26.6% | 0.032% |  | 0.63 | 1.96 |
| 4-6 | 10.6% | 0.017% |  | 50.9% | 0.015% |  | 0.78 | 2.50 |
|  |  |  |  |  |  |  |  |  |
| 2. 20-40 year |  |  |  |  |  |  |  |  |
| 1 | 29.7% | 0.069% |  | 34.7% | 0.045% |  | 0.19 | 0.53 |
| 2-3 | 13.9% | 0.023% |  | 24.6% | 0.040% |  | 0.66 | 1.53 |
| 4-6 | 12.0% | 0.012% |  | 21.7% | 0.030% |  | 0.95 | 2.03 |
|  |  |  |  |  |  |  |  |  |
| 3. 40-60 year |  |  |  |  |  |  |  |  |
| 1 | 33.1% | 0.072% |  | 26.2% | 0.065% |  | 0.25 | 0.50 |
| 2-3 | 7.9% | 0.020% |  | 23.4% | 0.043% |  | 1.00 | 2.61 |
| 4-6 | 5.4% | 0.016% |  | 11.8% | 0.048% |  | 1.29 | 2.97 |
|  |  |  |  |  |  |  |  |  |

Supplemental Table 9.A Proportions of Incident Diagnosis Treatment Combination (IDTC, 2011-2016) among those with and among those without Incident Dispensing of Antipsychotic medication (IDAP, 2011-2016), for migrants from sub-Saharan Africa and for native-born controls, by number of years since and age at arrival. The IRR of IDAP was re-estimated using these percentages.

|  | % IDTC among those with IDAP, native-born controls | % IDTC among those without IDAP, native-born controls |  | % IDTC among those with IDAP, migrants | % IDTC among those without IDAP, migrants |  | IRR of IDAP  Original | IRR of IDAP  Re-estimated |
| --- | --- | --- | --- | --- | --- | --- | --- | --- |
| All ages |  |  |  |  |  |  |  |  |
| Years since arrival 1 | 26.7% | 0.063% |  | 34.4% | 0.178% |  | 0.48 | 1.94 |
| 2-3 | 12.3% | 0.021% |  | 26.8% | 0.104% |  | 1.56 | 4.21 |
| 4-6 | 10.5% | 0.014% |  | 32.7% | 0.068% |  | 1.55 | 4.90 |
|  |  |  |  |  |  |  |  |  |
| Age at arrival |  |  |  |  |  |  |  |  |
| 1. 10-20 year |  |  |  |  |  |  |  |  |
| 1 | 5.8% | 0.023% |  | 37.5% | 0.108% |  | 0.33 | 4.80 |
| 2-3 | 9.9% | 0.015% |  | 38.7% | 0.072% |  | 1.44 | 5.33 |
| 4-6 | 10.6% | 0.017% |  | 52.9% | 0.017% |  | 1.55 | 4.69 |
|  |  |  |  |  |  |  |  |  |
| 2. 20-40 year |  |  |  |  |  |  |  |  |
| 1 | 29.7% | 0.069% |  | 35.5% | 0.210% |  | 0.54 | 2.15 |
| 2-3 | 13.9% | 0.023% |  | 22.5% | 0.132% |  | 1.67 | 4.27 |
| 4-6 | 12.0% | 0.012% |  | 22.6% | 0.107% |  | 1.63 | 5.30 |
|  |  |  |  |  |  |  |  |  |
| 3. 40-60 year |  |  |  |  |  |  |  |  |
| 1 | 33.1% | 0.072% |  | 33.3% | 0.168% |  | 0.46 | 1.35 |
| 2-3 | 7.9% | 0.020% |  | 29.4% | 0.035% |  | 1.36 | 3.28 |
| 4-6 | 5.4% | 0.016% |  | 40.0% | 0.000% |  | 1.65 | 4.45 |
|  |  |  |  |  |  |  |  |  |

Supplemental Table 9.B Proportions of Incident Diagnosis Treatment Combination (IDTC, 2011-2016) among those with and among those without Incident Dispensing of Antipsychotic medication (IDAP, 2011-2016), for migrants from non-Western countries other than sub-Saharan Africa and for native-born controls, by number of years since and age at arrival. The IRR of IDAP was re-estimated using these percentages.

|  | % IDTC among those with IDAP, native-born controls | % IDTC among those without IDAP, native-born controls |  | % IDTC among those with IDAP, migrants | % IDTC among those without IDAP, migrants |  | IRR of IDAP  Original | IRR of IDAP  Re-estimated |
| --- | --- | --- | --- | --- | --- | --- | --- | --- |
| All ages |  |  |  |  |  |  |  |  |
| Years since arrival 1 | 26.7% | 0.063% |  | 28.5% | 0.044% |  | 0.24 | 0.56 |
| 2-3 | 12.3% | 0.021% |  | 23.2% | 0.039% |  | 0.90 | 1.81 |
| 4-6 | 10.5% | 0.014% |  | 21.3% | 0.026% |  | 1.17 | 2.19 |
|  |  |  |  |  |  |  |  |  |
| Age at arrival |  |  |  |  |  |  |  |  |
| 1. 10-20 year |  |  |  |  |  |  |  |  |
| 1 | 5.8% | 0.023% |  | 16.7% | 0.031% |  | 0.22 | 1.45 |
| 2-3 | 9.9% | 0.015% |  | 26.5% | 0.039% |  | 0.69 | 2.22 |
| 4-6 | 10.6% | 0.017% |  | 55.6% | 0.022% |  | 0.67 | 2.53 |
|  |  |  |  |  |  |  |  |  |
| 2. 20-40 year |  |  |  |  |  |  |  |  |
| 1 | 29.7% | 0.069% |  | 33.3% | 0.043% |  | 0.18 | 0.49 |
| 2-3 | 13.9% | 0.023% |  | 27.4% | 0.036% |  | 0.78 | 1.62 |
| 4-6 | 12.0% | 0.012% |  | 21.5% | 0.021% |  | 1.13 | 1.95 |
|  |  |  |  |  |  |  |  |  |
| 3. 40-60 year |  |  |  |  |  |  |  |  |
| 1 | 33.1% | 0.072% |  | 34.7% | 0.066% |  | 0.32 | 0.61 |
| 2-3 | 7.9% | 0.020% |  | 21.1% | 0.056% |  | 1.70 | 3.60 |
| 4-6 | 5.4% | 0.016% |  | 13.6% | 0.044% |  | 2.21 | 3.80 |
|  |  |  |  |  |  |  |  |  |

Supplemental Table 9.C Proportions of Incident Diagnosis Treatment Combination (IDTC, 2011-2016) among those with and among those without Incident Dispensing of Antipsychotic medication (IDAP, 2011-2016), for migrants from Eastern Europe and for native-born controls, by number of years since and age at arrival. The IRR of IDAP was re-estimated using these percentages.

|  | % IDTC among those with IDAP, native-born controls | % IDTC among those without IDAP, native-born controls |  | % IDTC among those with IDAP, migrants | % IDTC among those without IDAP, migrants |  | IRR of IDAP  Original | IRR of IDAP  Re-estimated |
| --- | --- | --- | --- | --- | --- | --- | --- | --- |
| All ages |  |  |  |  |  |  |  |  |
| Years since arrival 1 | 26.7% | 0.063% |  | 34.4% | 0.043% |  | 0.17 | 0.54 |
| 2-3 | 12.3% | 0.021% |  | 25.7% | 0.037% |  | 0.59 | 1.55 |
| 4-6 | 10.5% | 0.014% |  | 20.4% | 0.039% |  | 0.84 | 2.19 |
|  |  |  |  |  |  |  |  |  |
| Age at arrival |  |  |  |  |  |  |  |  |
| 1. 10-20 year |  |  |  |  |  |  |  |  |
| 1 | 5.8% | 0.023% |  | 25.0% | 0.024% |  | 0.17 | 1.52 |
| 2-3 | 9.9% | 0.015% |  | 18.8% | 0.012% |  | 0.35 | 0.77 |
| 4-6 | 10.6% | 0.017% |  | 37.5% | 0.020% |  | 0.73 | 1.99 |
|  |  |  |  |  |  |  |  |  |
| 2. 20-40 year |  |  |  |  |  |  |  |  |
| 1 | 29.7% | 0.069% |  | 42.9% | 0.041% |  | 0.15 | 0.49 |
| 2-3 | 13.9% | 0.023% |  | 27.7% | 0.039% |  | 0.57 | 1.47 |
| 4-6 | 12.0% | 0.012% |  | 22.4% | 0.035% |  | 0.82 | 2.08 |
|  |  |  |  |  |  |  |  |  |
| 3. 40-60 year |  |  |  |  |  |  |  |  |
| 1 | 33.1% | 0.072% |  | 19.0% | 0.064% |  | 0.17 | 0.42 |
| 2-3 | 7.9% | 0.020% |  | 24.4% | 0.042% |  | 0.76 | 2.32 |
| 4-6 | 5.4% | 0.016% |  | 9.5% | 0.077% |  | 0.91 | 3.64 |
|  |  |  |  |  |  |  |  |  |

Supplemental Table 9.D Proportions of Incident Diagnosis Treatment Combination (IDTC, 2011-2016) among those with and among those without Incident Dispensing of Antipsychotic medication (IDAP, 2011-2016), for migrants from Western countries other than Eastern Europe and for native-born controls, by number of years since and age at arrival. The IRR of IDAP was re-estimated using these percentages.

|  | % IDTC among those with IDAP, native-born controls | % IDTC among those without IDAP, native-born controls |  | % IDTC among those with IDAP, migrants | % IDTC among those without IDAP, migrants |  | IRR of IDAP  Original | IRR of IDAP  Re-estimated |
| --- | --- | --- | --- | --- | --- | --- | --- | --- |
| All ages |  |  |  |  |  |  |  |  |
| Years since arrival 1 | 26.7% | 0.063% |  | 19.0% | 0.022% |  | 0.19 | 0.30 |
| 2-3 | 12.3% | 0.021% |  | 15.8% | 0.029% |  | 0.38 | 0.97 |
| 4-6 | 10.5% | 0.014% |  | 19.0% | 0.017% |  | 0.53 | 1.14 |
|  |  |  |  |  |  |  |  |  |
| Age at arrival |  |  |  |  |  |  |  |  |
| 1. 10-20 year |  |  |  |  |  |  |  |  |
| 1 | 5.8% | 0.023% |  | 0.0% | 0.004% |  | 0.16 | 0.17 |
| 2-3 | 9.9% | 0.015% |  | 7.7% | 0.022% |  | 0.29 | 0.82 |
| 4-6 | 10.6% | 0.017% |  | 50.0% | 0.000% |  | 0.41 | 1.04 |
|  |  |  |  |  |  |  |  |  |
| 2. 20-40 year |  |  |  |  |  |  |  |  |
| 1 | 29.7% | 0.069% |  | 25.0% | 0.021% |  | 0.16 | 0.28 |
| 2-3 | 13.9% | 0.023% |  | 15.5% | 0.028% |  | 0.36 | 0.87 |
| 4-6 | 12.0% | 0.012% |  | 20.0% | 0.017% |  | 0.53 | 1.13 |
|  |  |  |  |  |  |  |  |  |
| 3. 40-60 year |  |  |  |  |  |  |  |  |
| 1 | 33.1% | 0.072% |  | 12.5% | 0.044% |  | 0.22 | 0.30 |
| 2-3 | 7.9% | 0.020% |  | 27.3% | 0.033% |  | 0.41 | 1.61 |
| 4-6 | 5.4% | 0.016% |  | 0.0% | 0.028% |  | 0.65 | 1.12 |
|  |  |  |  |  |  |  |  |  |
